# Plasticity of Human Meniscus Fibrochondrocytes: A Study on Effects of Mitotic Divisions and Oxygen Tension

**DOI:** 10.1038/s41598-017-12096-x

**Published:** 2017-09-22

**Authors:** Yan Liang, Enaam Idrees, Stephen H. J. Andrews, Kirollos Labib, Alexander Szojka, Melanie Kunze, Andrea D. Burbank, Aillette Mulet-Sierra, Nadr M. Jomha, Adetola B. Adesida

**Affiliations:** 1 University of Alberta, Department of Surgery, Divisions of Orthopaedic Surgery and Surgical Research, Edmonton, T6G 2E1 Canada; 2 Division of Burn and Reconstructive Surgery, Second Affiliated Hospital, Shantou University Medical College, Shantou, Guangdong, People’s Republic of China

## Abstract

Meniscus fibrochondrocytes (MFCs) may be the optimal cell source to repair non-healing meniscus injuries using tissue engineering strategies. In this study, we investigated the effects of mitotic divisions and oxygen tension on the plasticity of adult human MFCs. Our assessment techniques included gene expression, biochemical, histological, and immunofluorescence assays. MFCs were expanded in monolayer culture with combined growth factors TGFβ1 and FGF-2 (T1F2) under normoxia (21% O_2_). Trilineage (adipogenesis, chondrogenesis and osteogenesis) differentiation was performed under both normoxic (21% O_2_) and hypoxic (3% O_2_) conditions. The data demonstrated that MFCs with a mean total population doubling of 10 can undergo adipogenesis and chondrogenesis. This capability was enhanced under hypoxic conditions. The MFCs did not undergo osteogenesis. In conclusion, our findings suggest that extensively expanded human MFCs have the capacity to generate tissues with the functional matrix characteristics of avascular meniscus. To this end, expanded MFCs may be an ideal cell source for engineering functional constructs for the replacement or repair of avascular meniscus.

## Introduction

Musculoskeletal diseases including osteoarthritis (OA) comprise an increasing proportion of the global burden of disease; in 2015, they were estimated to account for 6.7% of the global disability-adjusted life years, making them the fourth greatest burden on the health of the world’s population (third in developed countries)^1^. It is estimated that the cumulative economic burden of OA in Canada from 2010 to 2015 was $195 billion and annual costs are expected to rise in the future^2^.

Symptomatic OA of the knee affects over 10% of adults over the age of 60^3^. Injury to the knee menisci is a significant risk factor in the development of knee OA^4^. The knee menisci are load-bearing fibrocartilages positioned between the articular surfaces of the femoral condyle and tibial plateau. The menisci are integral to joint homeostasis by decreasing contact stresses^5^, increasing stability^6^ and aiding joint lubrication^7^. These complex functions are facilitated by the extracellular matrix (ECM), which is produced and maintained by a heterogenous population of cells in the menisci, the predominance of which are referred to as meniscus fibrochondrocytes (MFCs)^8^. This family of ECM molecules include an abundance of type I collagen throughout the meniscus, with substantial amounts of type II collagen and aggrecan in the avascular region^9–11^. The inner two-thirds of meniscus are colloquially termed the “white zone” because that area is avascular, receiving nutrients mainly by diffusion^12,13^. The avascular nature of this region combined with the severe loading within the knee joint inhibits its ability to repair. Current treatments for damaged avascular menisci have poor long-term outcomes with limited reduction in the incidence of OA progression^14^.

Due to the unsatisfactory outcomes of current treatments, cell-based tissue engineering (TE) strategies have been an area of interest for meniscus repair or replacement^15^. Meniscus TE aims to recreate meniscus-like tissue to replace damaged tissues after injury and restore normal function. Cell source is an important consideration for meniscus TE. The cell sources may include MFCs, articular chondrocytes, and precursor cells such as mesenchymal stem cells (MSCs). Previous research predominantly focused on articular chondrocytes^16,17^ and MSCs derived from bone marrow^18^, synovium^19^, and adipose tissue^20^. However, current work focuses on MFCs as a preferred cell source for two main reasons. First, they are derived from native meniscus tissue and are conditioned to synthesize the functional ECM of meniscus^8^. Second, when compared to the most commonly-used bone marrow-derived MSCs, MFCs were reported to have better fibrochondrogenic differentiation potential^21,22^ and form thicker collagen fibres and orientations resembling native meniscus^23^. MFCs were also demonstrated to have less tendency to form bone precursors through hypertrophic differentiation *in vitro* ^22^ and calcify *in vivo* ^24^. However, acquiring sufficient numbers of MFCs from surgically removed tissues to develop a TE meniscus replacement remains a major challenge. MFCs reside in a dense ECM *in situ* and account for only about 0.1–0.12% of the wet weight of normal meniscus^25^. Limited cell numbers are available to be isolated from meniscus tissue after partial meniscectomy, necessitating *in vitro* monolayer cell expansion^26^. However, MFCs were found to dedifferentiate and lose their matrix-forming phenotype after serial cell passaging^26–29^. Increased population doublings (PD) of MFCs resulted in significant downregulation of mRNA expression levels of type II collagen and aggrecan, with an increased gene expression of type I collagen^27,28^. Expanded MFCs were also shown to display trilineage differentiation plasticity^21^.

The combination of transforming growth factor β1 (TGFβ1) and fibroblast growth factor-2 (FGF-2) for cell expansion has been shown to promote proliferation rates of periosteal cells^30^ and human articular chondrocytes^31,32^. Furthermore, after expansion in TGFβ1 and FGF-2 both periosteal cells and articular chondrocytes demonstrated enhanced chondrogenic differentiation and restoration of the matrix-forming capacity, respectively. Moreover, oxygen tension was shown to improve matrix-forming phenotype of expanded MFCs especially after expansion with FGF-2^33^. Our previous work^34^ has shown that the hypoxia (5% O_2_) could also be beneficial for MFC proliferation and upregulation of the expression of collagen II and aggrecan in expanded MFCs with the use of FGF-2. However, little is known about the effect of combined TGFβ1 and FGF-2 (T1F2) on MFC proliferation and subsequent redifferentiation capacity under different oxygen tensions.

To this end, our objectives were to characterize the maximal population doublings (PD) for T1F2-expanded MFCs while retaining their functional matrix-forming capacity. The effect of oxygen tension (normoxia 21% O_2_, hypoxia 3% O_2_) on chondrogenic differentiation and matrix-forming phenotype of T1F2-expanded MFCs was also tested. We also characterized the adipogenic and osteogenic differentiation potential of these T1F2-expanded MFCs.

## Results

### Cell yield and expansion

**Figure 1:**
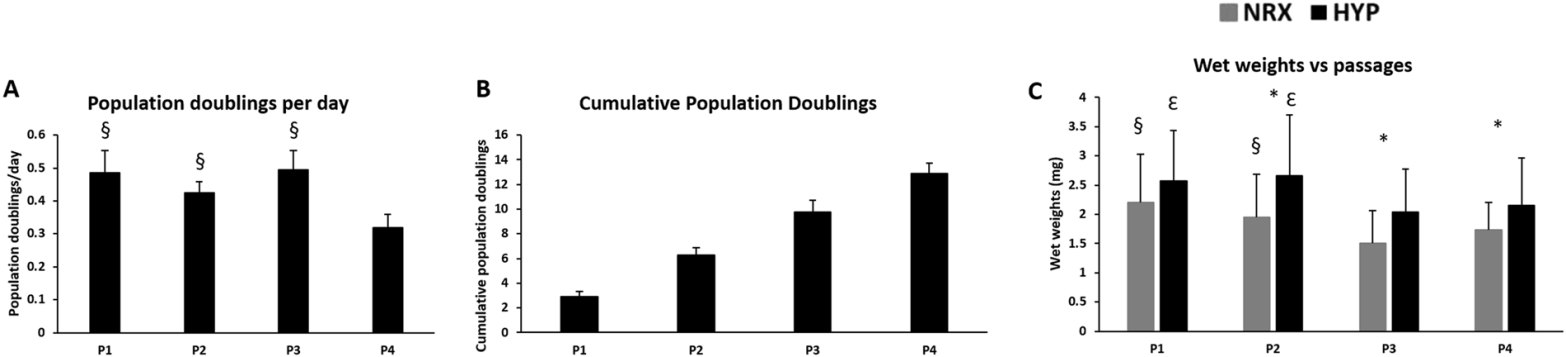
Isolation/expansion of meniscus fibrochondrocytes (MFCs) with TGFβ1 and FGF-2 (T1F2) for four passages under normoxia (NRX). (**A**) Population doublings per day (n = 6). Statistical analysis is presented as follows: ^§^Significance between P1-P3 and P4 in NRX (*p* < 0.05). P: passage; (**B**) Cumulative population doublings at each passage during expansion of MFCs (n = 6). (**C**) Wet weights of pellets derived from T1F2-expanded MFCs of four passages after 21 days chondrogenic stimulation under normoxia or hypoxia (HYP) (n = 6). Statistical analysis is presented as: ^§^significance between P1/P2 and P3 in NRX, ^ε^significance between P1/P2 and P3 in HYP, ^*^significance between NRX and HYP within the same passage (*p* < 0.05). Data all presented as mean ± standard deviation.

Meniscus tissues were obtained from 6 male donors (age: 20–37 years) undergoing partial meniscectomy for acute traumatic injury. Mean wet weight (±SD) of the meniscus tissue pre-digestion was 2.70 ± 0.87 g and mean viable cell yield (±SD) after 48 hours of post collagenase isolation culture was 3.14 ± 1.46 million cells/g of wet meniscus tissue. Isolated MFCs were cultured in monolayer with T1F2 under normal oxygen tension (21% O_2_). The cell morphologies were elongated fibroblast-like and small round-shaped chondrocyte-like during these 48 hours. After one week of monolayer culture with T1F2, the morphology of the cells became universally elongated and spindle-like. Mean population doublings (PD) per day (±SD) was 0.49 ± 0.07 at P1, 0.42 ± 0.04 at P2, 0.49 ± 0.06 at P3, decreasing to 0.32 ± 0.04 at P4. While P1-P3 were not significantly different from each other, P4 was significantly lower than each of the previous three passages (P1-P3 vs. P4 all *p* < 0.05) (Fig. 1A). The mean cumulative PD in monolayer culture (±SD) increased from 2.91 ± 0.41 at P1, 6.30 ± 0.57 at P2, 9.76 ± 0.96 at P3, and 12.89 ± 0.81 at P4 (Fig. 1B).

### Wet weights

At the end of each passage, 5 × 10^5^ MFCs were centrifuged into pellets for culture in a defined serum-free chondrogenic media containing TGFβ3. After 21 days of culture in normoxia (NRX, 21% O_2_) and hypoxia (HYP, 3% O_2_), wet weights of pellets were recorded as an indicator of ECM production (Fig. 1C). HYP resulted in increased wet weights when compared to NRX within each passage. While this difference was not significant in P1 (*p* = 0.145), it increased and became significant in P2 *(p* = 0.009), P3 (*p* = 0.006) and P4 (*p* = 0.037). The effect of passaging on wet weight was variable. A significant decrease was observed between P1 and P3 or P2 and P3 in both NRX and HYP (*p* < 0.05). However, this difference was not significant between P1 and P4 or P2 and P4 for both conditions (*p* > 0.05). There was a moderate-to-weak, but significant negative correlation (HYP: R^2^ = 0.34, adjusted p < 0.05, NRX: R^2^ = 0.26, adjusted p < 0.05) between wet weight and donor age when all passages were grouped. Interestingly, the correlation between age and wet weight when each passage was analyzed separately was stronger but not significant as passage increased for both HYP and NRX (i.e. HYP, P1: R^2^ = 0.20, P2: R^2^ = 0.35, P3: R^2^ = 0.56, P4: R^2^ = 0.69) (not shown).

### Biochemical analysis

**Figure 2:**
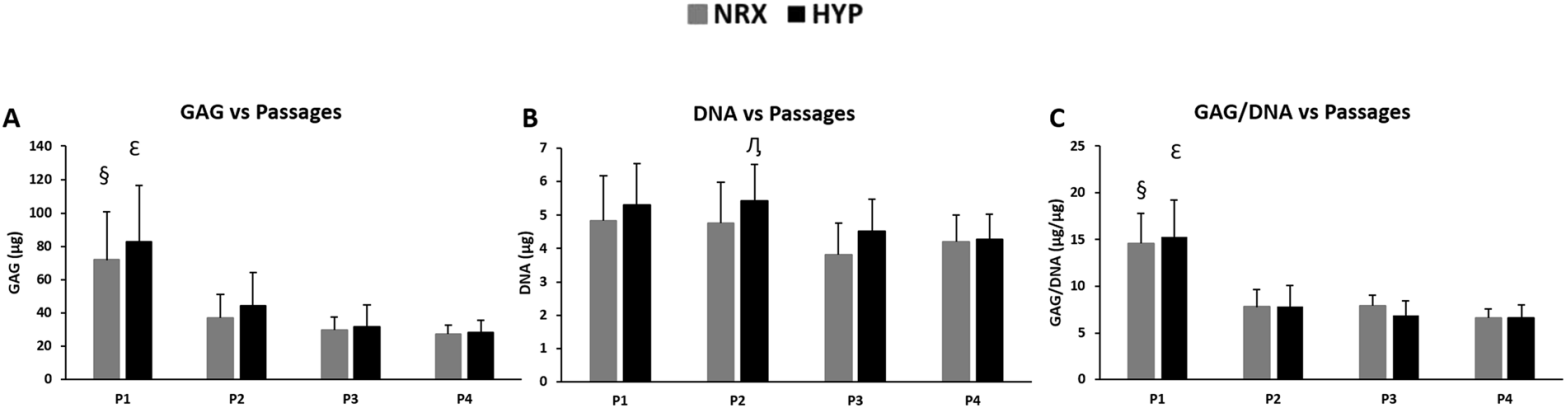
Biochemical analysis of pellets derived from T1F2-expanded MFCs of four passages after 21 days chondrogenic stimulation under NRX or HYP (n = 6). (**A**) GAG content, (**B**) DNA content and (**C**) GAG/DNA in pellets. Statistical analysis is presented as follows: ^§^Significance between P1 and P2-P4 in NRX, ^ε^significance between P1 and P2-P4 in HYP, ^η^significance between P2 and P4 in HYP (*p* < 0.05). Data all presented as mean ± standard deviation.

Biochemical analysis was performed to assess the glycosaminoglycan (GAG) and DNA contents in pellets after 21 days of culture in chondrogenic medium under NRX and HYP. GAG content was dramatically higher in P1 relative to subsequent passages in both oxygen tensions (Fig. 2A). It decreased significantly between P1 and P2-P4; with GAG content at P1 approximately double that of P2 (*p* = 0.004 NRX, *p* = 0.013 HYP) and highly significant when compared to P3 and P4 (all *p* < 0.001). A significant decrease was also observed between P2 and P4 in HYP only (*p* = 0.04). No other significant differences were observed in GAG content between passages. When GAG content was normalized to cellular DNA content (GAG/DNA), it followed the same trend (Fig. 2C). GAG/DNA decreased significantly between P1 and P2-P4 in both oxygen tensions; P1 was almost double when compared to P2 (*p* < 0.001 NRX, *p* = 0.0001 HYP), P3 (*p* = 0.001 NRX, *p* < 0.001 HYP) and P4 (NRX/HYP, all *p* < 0.001). There were no significant differences in GAG/DNA between P2-P4 under both oxygen tensions. Although there was a significant difference in DNA content in P2 compared to P4 in HYP, no other significant differences were found in DNA content within passages between oxygen tensions or between passages (Fig. 2B). No significant differences were found in GAG content or GAG/DNA between oxygen tensions within the same passage (Fig. 2A,C).

### Safranin-O Staining

**Figure 3:**
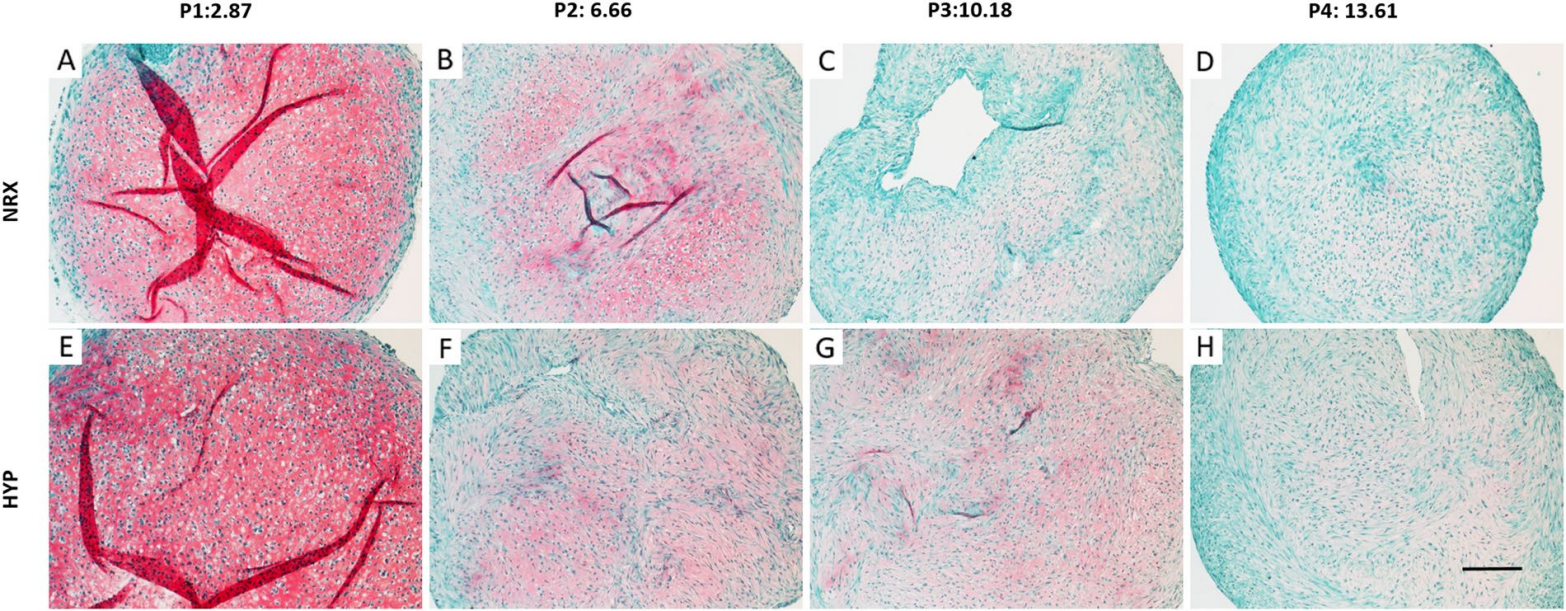
Safranin-O staining analysis for proteoglycan deposition in pellets derived from T1F2-expanded MFCs of four passages after 21 days chondrogenic stimulation under NRX or HYP from one representative donor (male, 20 years old). (**A**–**D**) Pellets cultured under NRX, (**E**–**H**) Pellets cultured under HYP. The top panel of numbers indicate population doublings of each passage. Scale bar: 200 µm.

After 21 days of culture in chondrogenic media, pellets from 4 passages were embedded, cut and stained with Safranin-O for proteoglycan deposition (pink/red staining). One representative donor (male, age 20) is presented in figures below. In both oxygen tensions, Safranin-O positive proteoglycan (pink staining) was most intense in P1, sharply decreasing in P2, and gradually decreasing to faint staining in P4. Chondrocyte-like (i.e. rounded) cells were in the lacunae-like structures in P1, but became progressively interspersed with fibroblast-like (i.e. elongated) cells from P2-P4 with loss of lacunae (Fig. 3).

Within P1 and P2, no qualitative differences were observed between oxygen tensions (Fig. 3A,B vs E,F). However, it appears HYP resulted in qualitatively more Safranin-O staining in P3 and P4 (Fig. 3G,H vs C,D). More chondrocyte-like cells in P3 were observed in HYP compared to NRX. Additionally, although pellets were cut to approximately the same depths, pellet diameter was qualitatively larger in HYP compared to NRX within the same passage, which is consistent with the differences in wet weights between oxygen tensions (Figs 1C & 3). Although proteoglycan production in HYP appeared to be retained in P3-P4 as compared to NRX qualitatively (pink staining under Safranin-O), quantitative analysis did not show significant differences to support this observation (Total GAG and GAG/DNA; Figs 2A,C vs. 3).

### Immunofluorescence

**Figure 4:**
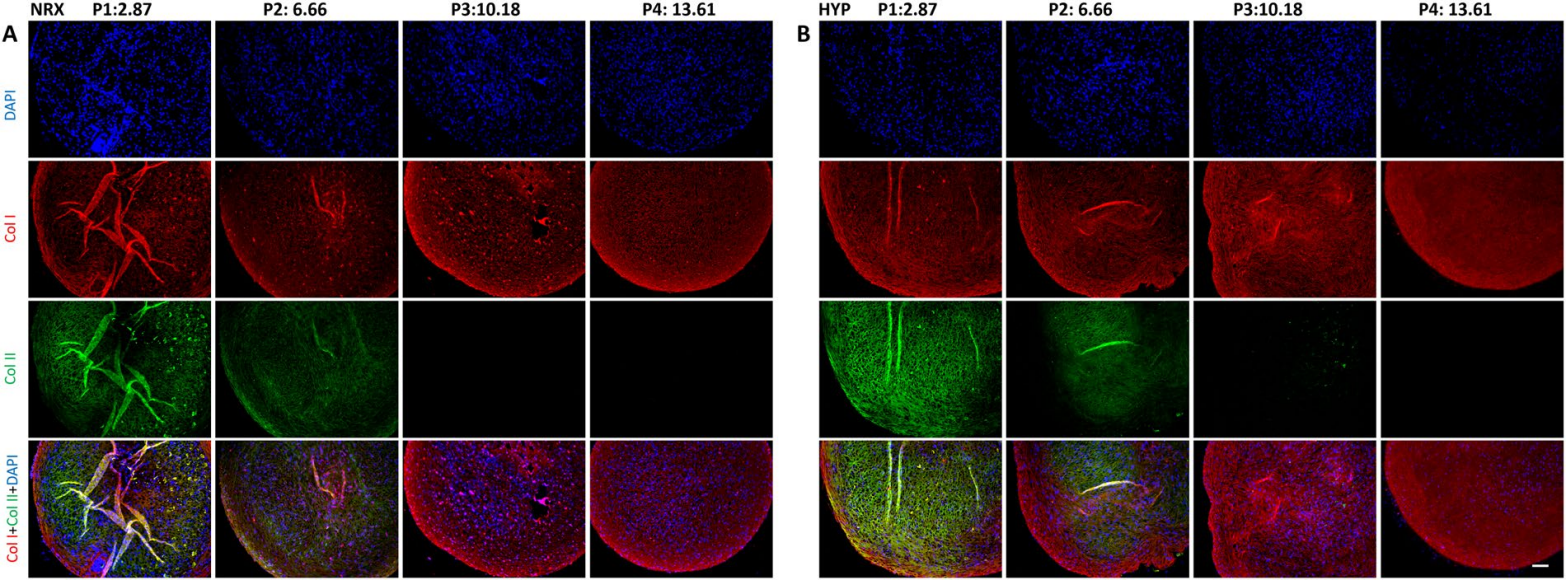
Immunofluorescence analysis of collagen I and collagen II in pellets derived from T1F2-expanded MFCs of four passages after 21 days chondrogenic stimulation under NRX or HYP from one representative donor (male, 20 years old). Blue (DAPI): cells, Red (Texas Red): collagen I, Green (FITC): collagen II. (**A**) Pellets cultured under NRX, (**B**) Pellets cultured under HYP from four passages. Scale bar: 100 µm

**Figure 5:**
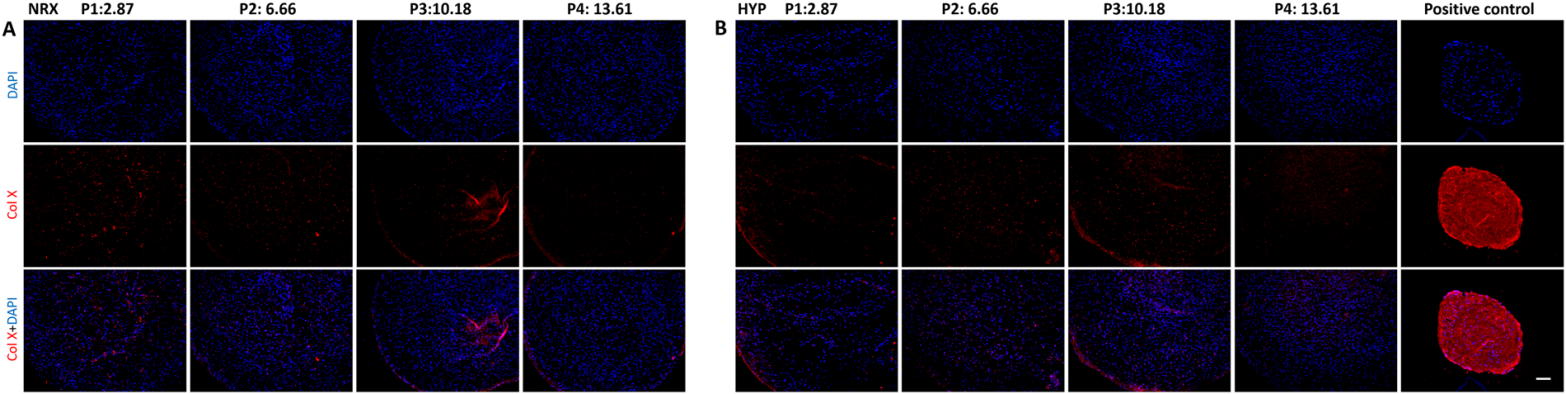
Immunofluorescence analysis of hypertrophic marker collagen X in pellets derived from T1F2-expanded MFCs of four passages after 21 days chondrogenic stimulation under NRX or HYP from one representative donor (male, 20 years old). Blue (DAPI): cells, Red (Texas Red): collagen X. (**A**) Pellets cultured under NRX, (**B**) Pellets cultured under HYP from four passages. Positive control: pellets from human bone marrow-derived mesenchymal stem cells cultured with the same chondrogenic medium under normoxic condition. Scale bar: 100 µm.

Indirect immunofluorescence was performed to detect cells (DAPI), extracellular matrix components using primary antibodies to collagen I and collagen II (Fig. 4) and the hypertrophic chondrogenic differentiation marker, collagen X (Fig. 5). Chondrogenically differentiated pellets (2.5 × 10^5^) of human bone marrow derived-mesenchymal stem cells (hBM-MSCs) under NRX served as a positive control for collagen X. Qualitatively, MFCs visualized via DAPI were evenly distributed throughout the pellets across all passages under both oxygen tensions. Collagen I was homogeneously distributed across passages. In contrast, collagen II immunofluorescence was most intense in P1 and then decreased in P2 in both oxygen tensions. Type II collagen immunofluorescence was only positive in HYP at P3 with ~10PDs but negative at P4 with ~13PDs with negligible presence in P3-P4 in both oxygen tensions. Collagen II immunofluorescence results corresponded well with Safranin-O staining for proteoglycan content. Negligible deposition of collagen X was observed by immunofluorescence in pellets derived from T1F2-expanded MFCs when compared to the pellets derived from hBM-MSCs (Fig. 5). Only punctate pericellular fluorescence was observed in MFC pellets compared to the diffuse fibrillar signal from the positive control hBM-MSC pellets.

### Gene expression analysis

#### Chondrogenesis

**Figure 6:**
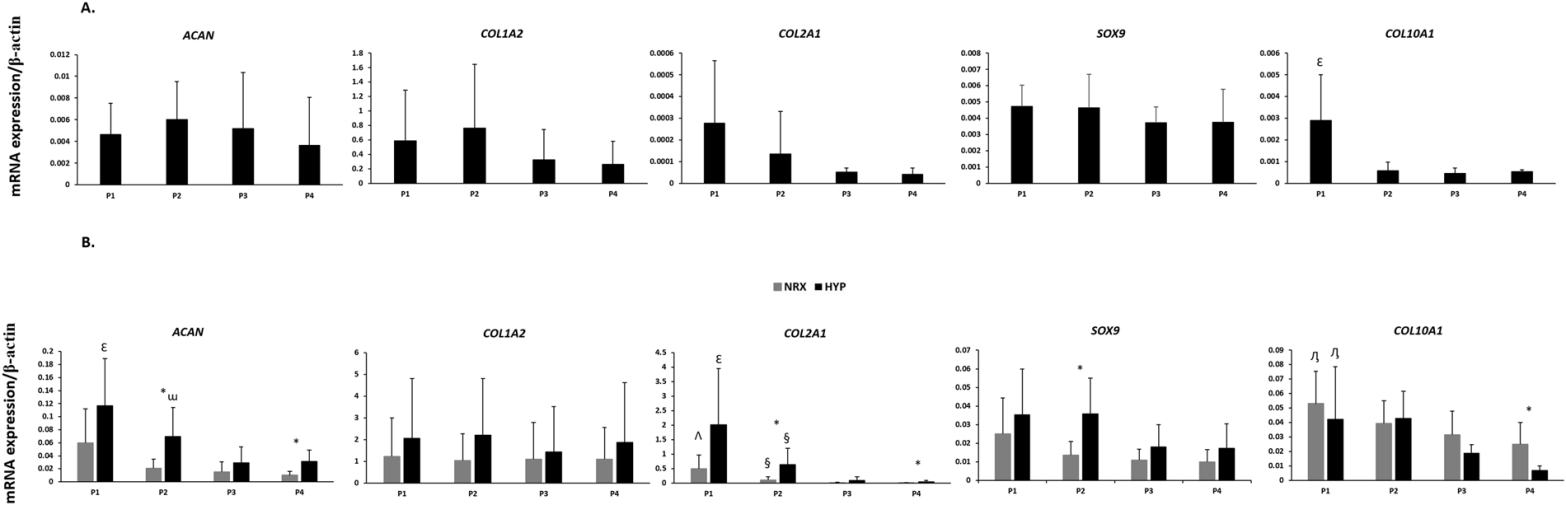
(**A**) Real-time PCR analysis of cDNA of T1F2-expanded MFCs in monolayer culture at the end of each passage prior to pellet culture (n = 4). ^ε^significance between P1 and P3/P4 (*p* < 0.05), (**B**) Real-time PCR analysis of cDNA of pellets derived from T1F2-expanded MFCs of four passages after chondrogenic stimulation for 21 days under NRX and HYP (n = 4). ^ε^Significance between P1 and P3/P4 in HYP, ^ω^significance between P2 and P3, ^^^significance between P1 and P3/P4 in NRX, ^§^significance between P2 and P3/P4 in both oxygen tensions, ^η^significance between P1 and P4 in both oxygen tensions, *significance between oxygen tensions within the same passage (*p* < 0.05). Data all presented as mean ± standard deviation.

To further characterize the ECM generated in pellets, gene expression was assessed by qRT-PCR after 21 days of chondrogenic culture under NRX or HYP. Additionally, similar gene expression analysis was conducted for monolayer cultured MFCs prior to centrifugation into pellets. The mean relative gene expression levels of aggrecan (*ACAN*), collagen I (*COL1A2*), collagen II (*COL2A1*), collagen X (*COL10A1*) and *SOX9* is presented in Fig. 6. In monolayer cultured MFCs with T1F2 (Fig. 6A), the overall trend for *ACAN* and *COL1A2* tended to decrease after P2 and *COL2A1* seemed to decrease from P1. The increased PD had no significant effects on gene expression levels of *ACAN*, *COL1A2*, *COL2A1* and *SOX9*. The relative gene expression level of *COL10A1* had a significant decrease in monolayer cultured MFCs from P1 to P3 (*p* = 0.009) and P1 to P4 (*p* = 0.014) although the relative levels were low.

In pellets (Fig. 6B), the relative gene expression level of *COL1A2* was not affected by increased PD or different oxygen tensions, which remained stable with no significant changes. Although relative gene expression levels of *ACAN*, *COL2A1* and *COL10A1* in pellets tended to decrease over passages, this change was not significant from P1-P2 in either oxygen tension. *ACAN* decreased significantly after serial passaging in HYP (P1 to P3 *p* = 0.008, P1 to P4 *p* = 0.02; P2 to P3 *p* = 0.046) while no significant changes were observed in NRX. *COL2A1* decreased significantly in pellets after serial passaging in both oxygen tensions (P1 to P3 and P1 to P4; P2 to P3 and P2 to P4, all *p* < 0.05). Gene expression levels of *SOX9* followed the same decreasing trend as *COL2A1*, but no significant difference was found between different passages. Gene expression levels of the hypertrophic marker *COL10A1* in pellets decreased significantly after serial passaging in both oxygen tensions (P1 to P4, *p* = 0.048 NRX/*p* = 0.001 HYP) with low relative expression levels.

Within the same passage, HYP tended to stimulate higher relative gene expression levels of *ACAN*, *COL1A2*, *COL2A1* and *SOX9* in pellets when compared to NRX (Fig. 6B). However, this trend was not significantly different in relative gene expression level of *COL1A2*. The relative gene expression level of *ACAN* was significantly higher in HYP than NRX of P2 (3.31-fold, *p* = 0.018) and P4 (3.07-fold, *p* = 0.01) while HYP stimulated a higher relative gene expression level of *COL2A1* in P1 (4.03-fold, approaching significance: *p* = 0.07), P2 (6.17-fold, p = 0.029) and P4 (8.91 fold: *p* = 0.085). The only significantly higher relative gene expression level for *SOX9* was found in P2 in HYP compared to NRX (2.60 fold, *p* = 0.014). In contrast, NRX tended to upregulate the relative gene expression level of *COL10A1* compared to HYP except for P2. It was 1.68-fold higher in P3 (approaching significance: *p* = 0.067) and 3.68-fold in P4 (*p* = 0.032) in NRX compared to HYP.

#### Adipogenic and osteogenic differentiation (gene expression and histological) analysis

**Figure 7:**
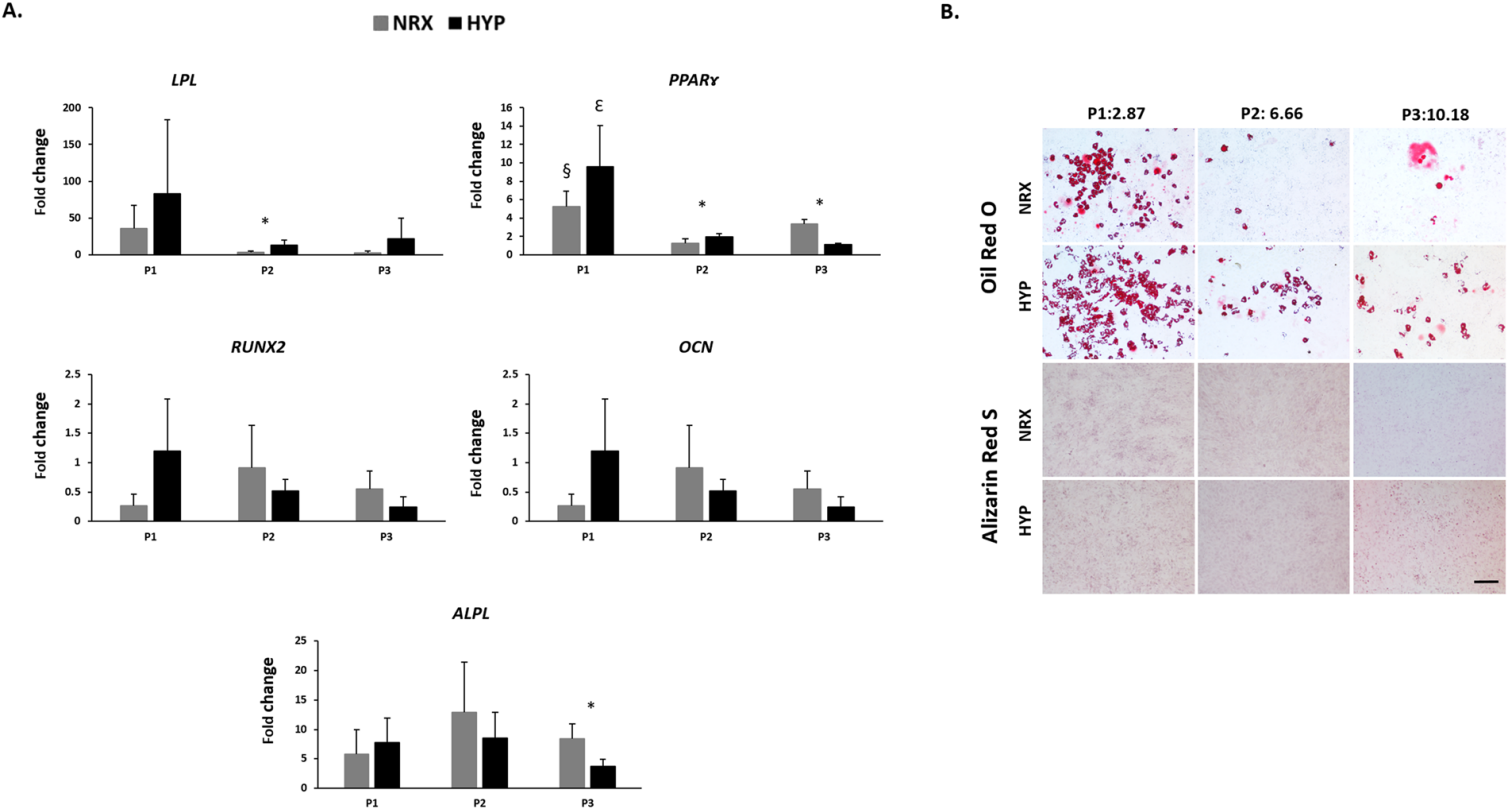
(**A**) Real-time PCR analysis of cDNA of T1F2-expanded MFCs of three passages with or without adipogenic and osteogenic induction under NRX and HYP (n = 4) and the fold changes of relative mRNA expression levels of adipogenic/osteogenic markers. Fold changes were calculated as ratio of NRX (induction)/NRX (control), HYP (induction)/HYP (control) within the same passage. ^*^significance between NRX and HYP within the same passage, ^§^significance between P1 and P2 in NRX, ^ε^significance between P1 and P3 in HYP, (*p* < 0.05). All mRNA expression levels were relative to β-actin. Data all presented as mean ± standard deviation. (**B**) Oil Red O/Alizarin Red S staining analysis for lipid droplet and bone matrix deposition in T1F2-expanded MFCs from 3 passages after adipogenic and osteogenic stimulation under NRX and HYP from one representative donor (male, 20 years old). The top panel of numbers indicate population doublings of each passage. Scale bar: 200 µm.

To further characterize the adipogenic and osteogenic capacity of T1F2-expanded MFC, the fold changes of relative gene expression levels in adipogenic and osteogenic groups were compared against control groups (without adipogenic and osteogenic induction media) within the same oxygen tension and passage, e.g. P1 (NRX) induction vs P1 (NRX) control (Fig. 7A). For adipogenic differentiation, the relative gene expression levels of lipoprotein lipase (*LPL*) and peroxisome proliferator-activated receptor gamma (*PPAR*_γ_) were upregulated compared to control cells without induction (i.e. fold change > 1) (Fig. 7A). Within passages, the relative gene expression levels tended to be higher in MFCs cultured under HYP for *LPL* and *PPAR*_γ_, corresponding with histological findings (not shown). In P2, the fold changes of gene expression levels of *LPL* were significantly higher in HYP compared to NRX (*p* = 0.035), while the fold changes of relative gene expression levels of *PPAR*_γ_ were significantly higher in P2 (*p* = 0.047) under HYP and P3 (*p* = 0.0026) under NRX. The fold change of relative gene expression levels appeared to be highest in T1F2-expanded MFCs from P1 in both oxygen tensions which is consistent with the Oil Red O staining. No significant difference was found in fold change of relative gene expression levels of *LPL* between passages while in *PPAR*_γ_, it was significantly higher in P1 compared to P2 (NRX, *p* = 0.003) and P1 compared to P3 (HYP, *p* = 0.002).

For osteogenic differentiation, the relative gene expression levels of the osteogenic markers, runt-related transcription factor 2 (*RUNX2*) and osteocalcin (*OCN*) were not upregulated compared to control cells without induction (i.e. fold change < 1). However, the relative gene expression levels of alkaline phosphatase (*ALPL*) were upregulated for all passages and oxygen conditions compared to control cells (i.e. fold change > 1). Significant differences were only found between NRX and HYP in P3 (*p* < 0.05) for *ALPL*. These gene expression results are consistent with the histological findings that showed no positive staining of Alizarin Red S in osteogenically induced T1F2-expanded MFCs.

T1F2-expanded MFCs from P1, P2 and P3 underwent adipogenic and osteogenic culture for 23 days and 21 days, respectively under HYP and NRX. The same representative donor as above (male, age 20) is presented in Figure 7B. Oil Red O and Alizarin Red S staining were performed to assess the formation of lipid droplets and bone matrix. All 3 passages stained positively with Oil Red O indicating adipogenic induction (Fig. 7B). In both oxygen tensions, the number of lipid droplets seemed to be highest in P1. In P1, HYP induced more lipid droplet formation than NRX, and this trend continued in P2-P3. While lipid droplets sharply decreased after P1 in NRX, HYP retained some adipogenic differentiation capacity of T1F2-expanded MFCs. In contrast to chondrogenic and adipogenic differentiation, no Alizarin Red S staining was observed in all three passages (Fig. 7B).

## Discussion

In cell-based meniscus tissue engineering strategies, a major challenge is the low cell yield from typical partial meniscectomy biopsies. When expanded during monolayer culture to increase cell numbers, the matrix-forming capacity of MFCs has been shown to decrease significantly from primary MFCs^27,29^ with a morphology change to fibroblast-like cells, a process referred to as dedifferentiation^26^. The primary goals of this study were to: 1) investigate the proliferation rates and subsequent chondrogenic differentiation of TGFβ1 and FGF-2 (T1F2)-expanded MFCs under normal oxygen (21% O_2_) or low oxygen tension (3%); 2) identify the number of population doublings (PD) these cells can undergo while still maintaining the capacity to form meniscus-like extracellular matrix; 3) to characterize the plasticity of these cells in terms of multilineage differentiation in chondrogenesis, adipogenesis and osteogenesis.

Growth factor supplementation during monolayer cell expansion has a profound effect on matrix-forming phenotype of expanded cells from a variety of sources^16,34^. The capacity of FGF-2 to enhance MSC^35,36^ and articular chondrocyte^37,38^ proliferation rates and subsequent chondrogenic differentiation has been well demonstrated. FGF-2 can also upregulate the synthesis of collagen II and aggrecan in MFCs from osteoarthritic human knee joints^34^. Another important growth factor, TGFβ1, can also enhance the proliferation of articular chondrocytes^39^ and periosteal cells^30^. When TGFβ1 and FGF-2 were used in combination, they had a synergistic effect on both proliferation and chondrogenic differentiation of chondrocytes in human articular cartilage explants^32^. However, when these two growth factors were combined with PDGF-BB during monolayer expansion of MFCs, proliferation rates increased but without any beneficial effects on matrix production^16^.

Here, we examined the effect of T1F2 on proliferation and restoration of chondrogenic re-differentiation capacity of MFCs. MFCs proliferated well in T1F2 supplemented medium; 7.5 ± 1.3 days were required for the MFCs to reach 80–85% confluence in monolayer culture. PD per day was similar in P1, P2 and P3 and then decreased significantly at P4 (Fig. 1A). When compared to our previous study, MFCs in P4 divided faster in T1F2 medium than in control-expanded MFCs without growth factors (0.32 ± 0.04 vs. 0.14 ± 0.03 doublings/day) or FGF-2-expanded MFCs (0.22 ± 0.04 doublings/day) in P1^34^. Contrary to previous studies^27–29^, gene expression of ECM molecules were not statistically different between passages to approximately 13 populations doublings in monolayer cultured cells (Fig. 6A): no significant decreases of collagen II and aggrecan or increase of collagen I gene expression in monolayer cultured cells were observed. While this result suggests that T1F2 may maintain the gene expression profile of normal human MFCs in monolayer expansion, the gene expression profile of aggrecan and type II collagen suggests a decline albeit not statistically significant. Other possible explanations for this unexpected finding may be differences in sources of MFCs and expansion conditions compared to published literature. For example, MFCs were isolated from young bovine (1–2 weeks) meniscus^27,29^ and human meniscus (54–79 years)^28^ obtained from osteoarthritic knee joints with either no growth factors or FGF-2 alone added to the expansion medium.

During chondrogenic stimulation, T1F2-expanded MFCs showed a chondrogenic response in the pellet model when compared to monolayer MFCs regardless of the PD (Fig. 6). The response was much greater in P1 MFCs with a PD of 2.9 ± 0.4. We observed that T1F2-expanded MFCs in P1 expressed a superior functional matrix-forming phenotype than those with a higher PD in both normoxic and hypoxic conditions. Biochemical analysis showed that total GAG content and GAG content normalized to DNA was approximately two times higher in P1 pellets than pellets with PD range of 6.3 ± 0.6 (P2) to 12.9 ± 0.8 (P4) (Fig. 2A,C). These results were consistent with Jakob *et al*. who found that T1F2-expanded articular chondrocytes regained their chondrocytic phenotype under proper stimulation^32^. Further, Safranin-O staining for proteoglycan deposition verified the dramatic decrease in chondrogenic capacity from P1 to P2 (Fig. 3). Interestingly, relative gene expression levels of chondrogenic markers aggrecan and collagen II in pellets showed no significant decreases from P1 to P2 (Fig. 6B), but immunofluorescence analysis for collagen II deposition revealed a substantial qualitative decrease from P1 to P2 (Fig. 4). This may be due to the relatively late time-point selected for the gene expression analysis (21 days of chondrogenic differentiation). A time course study investigating gene expression and matrix changes during chondrogenic differentiation of the T1F2-expanded MFCs could be performed in the future to optimize culture times. In contrast, T1F2-expanded MFCs from P3 (9.8 ± 1 PD) and P4 (12.9 ± 0.8 PD) showed qualitative differences in the deposition of collagen I between oxygen tensions. Hypoxia cultured T1F2 expanded MFCs were positive for type II collagen, albeit with reduced intensity. Normoxia cultured T1F2 expanded MFCs were negative for type II collagen. These findings are consistent with the Safranin-O staining intensity in P3 and P4. This indicates that T1F2-expanded MFCs with ~10 PDs may be appropriate for tissue engineering of the avascular inner meniscus which exists in a more hypoxic microenvironment than its vascularized outer counterpart.

To date, no studies have assessed the effect of oxygen tension on matrix-forming capacity of T1F2-expanded MFCs derived from normal human menisci. Oxygen tension in the knee joint is hypoxic^40,41^; thus, several studies have investigated the effect of oxygen tension on chondrogenic differentiation of expanded MFCs. Adesida *et al*. showed that hypoxic conditions (5% O_2_) enhanced matrix-forming capacity of MFCs from OA knee joints^34^ and several others have demonstrated the positive effects of low oxygen tension on chondrogenic differentiation of BM-MSCs^42,43^. In our study, HYP (3% O_2_) enhanced the expression of aggrecan (*ACAN*) and collagen II (*COL2A1*) when compared to NRX (21% O_2_) in T1F2-expanded MFCs derived from non-arthritic knee joints (Fig. 6B). HYP culture stimulated a more intense Safranin-O staining in P3 and P4 (Fig. 3). This finding was consistent with the wet weight results (Fig. 1C), albeit with no significant difference in total GAG contents relative to NRX cultured pellets (Fig. 2A). It is probable that the total collagen content of the pellets differs between NRX and HYP; HYP has been reported to increase collagen content^44^ and collagen has been reported to hold water^45^. Taken together, chondrogenic culture under hypoxic conditions resulted in a more robust chondrogenic differentiation of T1F2-expanded MFCs, which may improve their clinical applicability for avascular meniscus tissue engineering.

In our study, adipogenic differentiation of human T1F2-expanded MFCs was observed under both hypoxic and normoxic conditions (Fig. 7B). T1F2-expanded MFCs derived from P1 exhibited superior adipogenic capacity under hypoxic conditions. Increased PD resulted in decreased staining for lipid droplets in both oxygen tensions, and adipogenic differentiation was benefited by hypoxic conditions in all three passages. In contrast, no osteogenesis was induced under either oxygen tension, demonstrated by the absence of Alizarin Red staining for deposition of calcium (Fig. 7B). This was consistent with Mauck *et al.* who demonstrated minimal deposition of bone matrix in bovine MFCs from the inner region of the meniscus. However, that study did demonstrate substantial osteogenic differentiation in cells obtained from the outer portion of the meniscus tissue^46^. Since the meniscus tissues in our study were obtained from partial meniscectomy, most of the tissues are likely to be from the inner, avascular region of meniscus. Gross observation of the meniscus tissues obtained also suggested that they were removed from the inner meniscus. The outer meniscus regions possess a spontaneous healing capacity, which may not only be due to sufficient blood supply but due to the presence of perivascular derived stem cells^47^. The absence of osteogenesis in our study may also be related to MFC phenotype after expansion in T1F2. Previous studies have shown that expanded MFCs from the whole meniscus of humans^22,48^ or animals^21,49,50^ have similar surface markers to MSCs and have multipotent differentiation capacity, including osteogenesis. However, the cells from those studies were expanded without growth factor supplementation which may have been a factor in the lack of osteogenesis observed in our study. Moreover, it is well known that the meniscus contains a heterogeneous cell population, which varies from inner to outer regions^8,51^, and by using the whole tissue the population of cells would be quite different. Recently, however, Fu *et al*. have shown that expanded human MFCs from the inner region did exhibit osteogenic differentiation, but again no growth factors were used during cell expansion^52^. This identifies that the use of T1F2 in inner meniscus cells may inhibit their capacity to undergo osteogenic differentiation but this requires further examination to gain mechanistic insight. Collagen X (*COL10A1*) is a marker of hypertrophic differentiation of MSCs and has been correlated with bone formation after chondrogenic stimulation both *in vitro* and *in vivo* ^24^. It was notable that negligible deposition of collagen X was observed in all pellets from T1F2-expanded human MFCs when compared to human BM-MSCs (Fig. 5). This lack of collagen X supports the findings of limited osteogenic potential in these expanded MFCs. Low relative gene expression levels of collagen X were demonstrated in pellets derived from T1F2-expanded MFCs and no significant differences were found between oxygen tensions (Fig. 6B). This is in contrast to other studies which have shown that hypoxic conditions can suppress hypertrophic differentiation of MSCs^43,53^. These results suggest that the T1F2-expanded MFCs may be a promising cell source for meniscus repair with a potentially stable chondrocytic phenotype without the tendency of hypertrophic differentiation *in vitro*. While a study comparing cell sources using matched donors may be of interest in determining their relative advantages for meniscus repair, the difficulty in obtaining multiple tissues from healthy donors for this purpose may be prohibitive. Further study will be required to investigate the phenotypic stability of T1F2-expanded MFCs *in vivo* to ensure they do not undergo hypertrophic differentiation and calcification, as well as adipogenesis.

To determine the cell density of surgically-removed meniscus tissue, viable primary cell yield per gram wet weight of meniscus tissue was calculated. MFC physiology changes in meniscus tissue in osteoarthritic knee joints^54^. For this reason, tissues in this study were obtained only from partial meniscectomy patients suffering from acute injuries to limit the effects of chronic injury on cell biology. A limitation of this study was that we did not have detailed information regarding the severity of damage to donor tissues and the precise portion of the meniscus they were taken from. However, in general partial meniscectomy removes the irreparable inner avascular regions, which would have influenced the phenotypes of the initial cell population. Donor age is another variable which has been demonstrated to be relevant for cell yield from articular cartilage^55^. In this study, we did not find significant age effects on cell yield; however, it should be noted that all donors were relatively young (20–37) and otherwise healthy. As a future study, it may be of interest to compare the cell yield and tissue quality formed by MFCs derived from patients from a wider age range. The average cell yield per gram of meniscus tissue in this study was lower than that previously shown in human articular cartilage in donors between 20–40 years old^55^ (3.14 ± 1.46 × 10^6^ cells/g vs 7.9 × 10^6^ cells/g). These results have a significant clinical implication. Based on the fact that the volume of human medial and lateral meniscus is approximately 4.50 cm^3^ and 4.95 cm^3^ respectively^56^, and the potentially optimal cell seeding density of a scaffold for meniscus tissue engineering is 5 × 10^6^ cm^3^ ^57^, a total of 22.5–25 million cells would be required to generate an entire human medial or lateral meniscus. Damaged meniscus tissue could be partially removed arthroscopically and used to isolate autologous MFCs. These could be expanded with T1F2 and seeded on scaffolds that mimic the natural meniscus environment. *In vitro* strategies to recapitulate the different regions of the meniscus could then be employed, such as using growth factor-releasing scaffolds^58^ and mechanical conditioning under hypoxia to generate tissue with meniscus-like composition and mechanical properties before implantation. We note that substantial work is needed to ensure the new tissues restore normal meniscus function in protecting the articular cartilage to prevent early onset osteoarthritis *in vivo*. In our study, T1F2-expanded MFCs with approximately 3.3 PD retained the best matrix-forming capacity without hypertrophic differentiation. After 3 PD, i.e. 8× the mean number of primary MFCs (3.14 ± 1.46 million/g) in monolayer expansion, approximately 25 million MFCs can be obtained from 1 g of meniscus tissue. If a meniscus biopsy has an insufficient cell yield and higher numbers of MFCs are needed, expansion can be continued to P2 (PD: ~7, i.e. 128x the number of primary MFCs) while retaining the capacity for proteoglycan and collagen II production, albeit in reduced quantities relative to P1. This may be clinically relevant for meniscus repair for older patients or particularly small meniscus biopsies less than 1 g.

## Conclusion

In this study, we have characterized the proliferation rates and chondrogenic capacity of TGFβ1 and FGF-2 (T1F2)-expanded meniscus fibrochondrocytes (MFCs) under normoxic and hypoxic conditions. We found that MFCs expanded up to 10 doublings have the capacity to express the extracellular matrix (ECM)-forming phenotype especially under hypoxic conditions which is consistent with their natural microenvironment with the knee joint^41^. MFCs in the range of 2.9 + 0.4 PDs synthesized the most glycosaminoglycans and the highest Safranin-O positive ECM. For the first time, we demonstrated that the T1F2 expansion strategy may produce enough cells possessing an ECM-forming phenotype to repair a meniscus defect from a small tissue biopsy within a relatively brief period of time based on the human meniscus volume and the optimal seeding density for a type I collagen scaffold. Hypoxia was shown to be advantageous for chondrogenic culture, resulting in improved ECM quality and relevant gene expression profiles of MFCs at low PDs. Furthermore, we have demonstrated that hypertrophic and osteogenic tendencies are virtually absent for MFCs expanded with T1F2, although they show adipogenic capacity. Overall, this highlights the potential use of T1F2-expanded MFCs from the inner meniscus in combination with hypoxic culture conditions to produce robust tissue engineered meniscus-like ECM. Further investigation will be required to evaluate the phenotypic stability of this cell source *in vivo* and to build grafts of clinically-relevant size on three dimensional scaffolds.

## Methods

### Ethics Statement

Experimental methods and tissue collection were with the approval of and in accordance the University of Alberta’s Health Research Ethics Board- Biomedical Panel (Study ID: Pro00018778). Ethics Board waived the need for written informed consent of patients, as specimens used in the study were intended for discard in the normal course of the surgical procedure. Extensive precautions were taken to preserve the privacy of the participants donating specimens.

### Isolation and expansion of human meniscus fibrochondrocytes (MFCs)

Fresh meniscus specimens were obtained from six male patients undergoing partial meniscectomy for acute traumatic injuries (ages 20–37, mean age 28 ± 6 years). Wet weights of meniscus tissue were recorded before collagenase mediated digestion for MFC isolation. MFCs were released via treatment with trypsin-EDTA (0.05% w/v; Corning, Mediatech Inc. VA, USA) at 37 °C for 1 hour followed by 22 hours at 37 °C in type II collagenase (0.15% w/v; 300 U/mg solid; Worthington, NJ, USA) in a high glucose Dulbecco’s modified Eagle’s medium (DMEM; 4.5 mg/mL D-Glucose) supplemented with 5% v/v fetal bovine serum (FBS) (all from Sigma-Aldrich Co., MO, USA). The cell suspension obtained after digestion was passed through a 100 µm nylon-mesh filter (Falcon, BD Bioscience, NJ, USA). Isolated cells were plated at 10^4^ cells/cm^2^ and cultured in a standard medium: high glucose DMEM supplemented with 10% FBS (Sigma-Aldrich), 100 U/mL penicillin, 100 μg/mL streptomycin, 2 mM L-glutamine, and 10 mM 4-(2-hydroxyethyl)-1-piperazineethanesulfonic acid (HEPES) (Sigma-Aldrich) (all others from Life Technologies, ON, Canada) for 48 hours under normal oxygen tension (~21% O_2_; 5% CO_2_/95% air) at 37 °C in a humidified incubator. After 48 hours (passage 0), non-adherent cells were aspirated and adherent primary cells were detached with trypsin-EDTA (0.05% w/v). Thereafter the number of viable MFCs were counted using a haemacytometer after trypan blue staining. MFCs were plated at 10^4^ cells/cm^2^ and cultured in the standard medium described above supplemented with FGF-2 (5 ng/mL; Neuromics, MN, USA, Catalog#: PR80001) and TGFβ1 (1 ng/mL; ProSpec, NJ, USA, Catalog#: cyt-716) under normal oxygen tension (21% O_2_) at 37 °C in a humidified incubator, as previously described^33,34^. When cells were 80–85% confluent, first-passage (P1) cells were detached with trypsin-EDTA and culture was continued at 10^4^ cells/cm^2^ to produce second passage (P2), third passage (P3) and fourth passage (P4) cells. MFCs at the end of each passage were counted and population doublings during exponential growth phase were calculated as log_2_(N/N_0_), where N_0_ is the number of cells plated at the beginning of a passage and N is the number of cells counted at the end of a passage^59^.

### Mitotic effects on MFC chondrogenic differentiation potential

Chondrogenic differentiation was performed by using a three-dimensional cell pellet culture model. At the end of each passage, 5 × 10^5^ of MFCs were centrifuged at 1500 rpm for 5 minutes to make pellets in 1.5 mL sterile conical microtubes with removable screw-type lids (Bio Basic Inc, Ontario, Canada). For each condition (i.e. for each passage, oxygen tension, and donor) six identical pellets were set up to provide two technical replicates for biochemical, histological and gene expression analysis. The pellets were then cultured in 0.5 mL of serum-free chondrogenic medium consisting of high glucose DMEM (Sigma-Aldrich) containing 100 U/mL penicillin, 100 μg/mL streptomycin, 2 mM L-glutamine, 10 mM HEPES (Sigma-Aldrich) (all others from Life Technologies), ITS + 1 premix (Corning, Discovery Labware, Inc., MA, USA), 10 ng/mL transforming growth factor β3 (TGFβ3; ProSpec, NJ, USA, Catalog#: cyt-113), 100 nM dexamethasone, 365 μg/mL ascorbic acid 2-phosphate, 125 µg/mL human serum albumin and 40 μg/mL L-proline (all from Sigma-Aldrich) under normal oxygen (NRX; 21% O_2_) or low oxygen tension (HYP; 3%) at 37 °C in a humidified incubator with 5% CO_2_ for 21 days, as previously described^53,60^. Conical microtube lids were loosened to allow gas exchange during culture. At the same time, 5 × 10^5^ cells were suspended in 1 mL of Trizol (Life Technologies) as a control for monolayer culture gene expression. Medium changes were performed twice per week in a normoxic environment and the pellets cultured in hypoxic conditions thus had a brief exposure to normoxic conditions (<5 minutes per media change). At the end of each culture stage, the wet weights of cell pellets were recorded and the pellets were assessed biochemically for glycosaminoglycan (GAG) and DNA content, histologically and immunofluorescence for cartilage-specific matrix proteins and by real time quantitative reverse transcription polymerase chain reaction (qRT-PCR) for gene expression analysis.

### Mitotic effects on MFC adipogenic differentiation potential

At the end of P1, P2, and P3, adipogenesis was performed by plating 5 × 10^3^ MFCs/cm^2^ from 4 donors in a six-well plate (Falcon, BD, NJ, USA) as previously described^60^, with three technical replicates for the induction group for each condition. Initially, MFCs were cultured in 3 mL of the standard medium supplemented with FGF-2 (5 ng/mL) and TGFβ1 (1 ng/mL) until confluent in each well under NRX. Adipogenesis was then induced in hypoxic or normoxic conditions for three days by adding 3 mL of the standard medium supplemented with 1 μM dexamethasone, 0.5 mL ITS + 1, 100 μM indomethacin, and 500 μM isobutyl-1-methylxanthine (IBMX; Sigma-Aldrich) and then by culturing the cells in 3 mL of the standard medium supplemented with 0.5 mL ITS + 1 for one day. This induction-culture cycle was repeated four times. Then, cells were cultured in 3 mL of the standard medium supplemented with 0.5 mL of ITS + 1 only for another 7 days. At the end of 23 days’ culture, the culture medium was removed and cells were collected by adding 1 mL of Trizol per well followed by total RNA extraction for gene expression by qRT-PCR analysis or fixed with 2 mL of 10% w/v buffered formalin (Anachemia Canada Co, QC, Canada) for 3 minutes, then stained with 3 mL of 0.3% w/v Oil Red O (Sigma-Aldrich) per well for 1 hour at room temperature. After Oil Red O was removed and the cells were washed with distilled water three times, the staining was examined immediately by taking pictures using an Eclipse Ti-S microscope (Nikon Canada, ON, Canada).

### Mitotic effects on MFC osteogenic differentiation potential

At the end of P1, P2, and P3, osteogenesis was performed by plating 5 × 10^3^ MFCs/cm^2^ from 4 donors in a six-well plate (Falcon, BD, NJ, USA) as previously described^60^, with three technical replicates for the induction group for each condition. Briefly, MFCs were cultured in 3 mL of osteogenic medium consisting of the standard medium supplemented with 100 μM ascorbic acid 2-phosphate, 10 nM dexamethasone, and 10 mM beta (β)-glycerophosphate (all from Sigma-Aldrich) for 21 days with medium changed twice per week. After 21 days’ culture, culture medium was removed and cells were collected by adding 1 mL of Trizol per well, followed by total RNA extraction for gene expression by qRT-PCR analysis or fixed with 2 mL of 10% w/v buffered formalin for 10 minutes and stained with 1 mL of 1% w/v Alizarin Red S (Sigma Aldrich) (pH = 4.2) for 30 minutes at room temperature followed by washing with distilled water for 1 hour on an orbital shaker. The staining was examined immediately by taking pictures using an Eclipse Ti-S microscope or preserved in 70% v/v glycerol (Fisher Scientific, NH, USA) at 4 °C.

### Histology for MFC chondrogenesis

After 21 days of chondrogenic culture, pellets were removed from medium, fixed overnight in 10% v/v neutral buffered formalin at 4 °C, dehydrated by serially-dipping into ethanol baths of increasing concentration and embedded in paraffin wax. 5 µm thick sections were cut and stained with 0.01% (w/v) Safranin-O and counterstained with 0.02% (w/v) fast green (Sigma-Aldrich) to reveal proteoglycan matrix deposition as described previously^33^.

### Biochemical analysis for MFC chondrogenesis

After 21 days of chondrogenic culture, pellets were rinsed in 500 µL of phosphate buffered saline (Sigma-Aldrich) to remove residual medium and were then digested in 250 µL of proteinase K (1 mg/mL in 50 mM Tris with 1 mM EDTA, 1 mM iodoacetamide, and 10 mg/mL pepstatin A; all from Sigma-Aldrich) overnight at 57 °C. The GAG content was measured spectrophotometrically after 1,9-dimethylmethylene blue binding using chondroitin sulfate as standard (Sigma-Aldrich)^61^. The DNA content was determined using the CyQuant cell proliferation assay Kit (Invitrogen, ON, Canada) with supplied bacteriophage λ DNA as standard.

### Immunofluorescence for MFC chondrogenesis

5 µm thick paraffin-embedded pellets were deparaffinized, rehydrated, and then treated with protease XXV (AP-9006-005, Thermo Scientific) and hyaluronidase (H6254, Sigma-Aldrich). Sections were then incubated with primary antibody: rabbit anti-collagen I (CL50111AP-1, Cedarlane, ON, Canada), mouse anti-collagen II (II-II6B3, Developmental Studies Hybridoma Bank, IA, USA) using a 1:200 dilution and rabbit anti-collagen X (58632, Abcam, UK) using 1:100 dilution at 4 °C overnight, followed by incubation with a goat anti-rabbit IgG (H&L Alexa Fluor 594, Abcam, UK) with a 1:200 dilution for collagen I, X and goat anti-mouse IgG (H&L Alexa Fluor 488, Abcam) with a 1:200 dilution for collagen II. Sections were then stained with DAPI (4′, 6-diamidino-2-phenylindole, Cedarlane) and mounted with Glycerol and PBS (1:1 ratio). Immunofluorescence was visualized by an Eclipse Ti-S microscope (Nikon Canada, Mississauga, Canada).

### Gene expression analysis of MFC trilineage differentiation

Total RNA was extracted from cell suspensions for adipogenesis, osteogenesis, and monolayer controls as well as cell pellets after grinding with Molecular Grinding Resin (G-Biosciences, MO, USA) for chondrogenesis using Trizol (Life Technologies). To reduce changes of gene expression levels, cell suspensions and pellets were transferred into Trizol immediately when harvesting. Total RNA (100 ng) in a 40 µL reaction was reverse transcribed to cDNA by GoScript reverse transcriptase using 1 µg of oligo(Dt) primers (all from Promega Corporation, WI, USA). Reverse-transcription quantitative polymerase chain reaction was performed in a DNA Engine Opticon I Continuous Fluorescence Detection System (Bio-RAD, CA, USA) using hot start Taq and SYBR Green detection (Eurogentec North America Inc, San Diego, CA, USA). Primers sequences were obtained from previously published work and purchased from Invitrogen (Supplementary information -Table 1). mRNA expression levels for each primer set were normalized to the expression level of β-actin using the 2^−^ct^ method^62^.

**Table 1:** Primer sequences used in quantitative polymerase chain reaction analysis.

Gene	Primer sequences		NCBI Reference
β-actin (*ACTB*)	5′AAGCCACCCCACTTCTCTCTAA3′	Forward	NM_0011101
	5′AATGCTATCACCTCCCCTGTGT3′	Reverse	
Aggrecan (*ACAN*)	5′AGGGCGAGTGGAATGATGTT 3′	Forward	M55172
	5′GGTGGCTGTGCCCTTTTTAC3′	Reverse	
Collagen I (*COL1A2*)	5′TTGCCCAAAGTTGTCCTCTTC T3′	Forward	NM_000089
	5′AGC TTCTGTGGAACC ATG GAA3′	Reverse	
Collagen II (*COL2A1*)	5′CTGCAAAATAAAATCTCGGTGTTCT3′	Forward	NM_033150
	5′GGGCATTTGACTCACACCAGT3′	Reverse	
Collagen X (*COL10A1*)	5′GAAGTTATAATTTACACTGAGGGTTTCAAA3′	Forward	X60382
	5′GAGGCACAGCTTAAAAGTTTTAAACA3′	Reverse	
SRY-Box 9 (*SOX9*)	5′GACTTCCGCGACGTGGAC3′	Forward	Z46629
	5′GTTGGGCGGCAGGTACTG3′	Reverse	
Lipoprotein Lipase (*LPL*)	5′TGTGGATGTGTAAATGGAGCTTGT3′	Forward	NM_000237
	5′CACATACAGTTAGCACCACACATTTATAA3′	Reverse	
Alkaline Phosphatase (A*LPL*)	5′CCTGGCAGGGCTCACACT3′	Forward	NM_000478
	5′AAACAGGAGAGTCGCTTCAGAGA3′	Reverse	
Peroxisome proliferative activated receptor, gamma (*PPARγ*)	5′AAGCTGCTCCAGAAAATGACAGA3′	Forward	NM_138712
	5′CGTCTTCTTGATCACCTGCAGTA3′	Reverse	
Osteocalcin (*OCN*)	5′AATCCGGACTGTGACGAGTTG3′	Forward	NM_199173
	5′CCTAGACCGGGCCGTAGAAG3′	Reverse	
Runt related transcription factor 2 (*RUNX2/CBFA1*)	5′GGAGTGGACGAGGCAAGAGTTT3′	Forward	NM_001024630
	5′AGCTTCTGTCTGTGCCTTCTGG3′	Reverse	

### Statistical analysis

Data are presented as mean ± standard deviation. Statistical analyses were performed by SPSS version 23 (IBM, NY, USA) and Excel 2016 (Microsoft, WA, USA). Normality of data was assessed with Shapiro Wilk test. Levene’s test was used to assess the equality of variance for variables before multiple comparisons. For cases with equal variances, different passage groups were compared using one-way analysis of variance (ANOVA) with Tukey’s multiple comparison *post hoc* tests within the same oxygen tension; otherwise a Kruskal-Wallis one-way ANOVA with pairwise comparisons was applied. For comparison between two oxygen tensions within the same passage, a Student’s *t*-test was used. Significance was considered when *p* < 0.05. Pearson’s correlation coefficient was determined to assess linear correlation between two variables.

## References

[1] Woolf AD (2015). Global burden of osteoarthritis and musculoskeletal diseases. BMC Musculoskeletal Disorders.

[2] Arthritis Alliance of Canada. The impact of arthritis in Canada: today and over the next 30 years, 2011).

[3] Zhang Y, Jordan JM (2010). Epidemiology of osteoarthritis. Clinics in geriatric medicine.

[4] Roos H, Adalberth T, Dahlberg L, Lohmander LS (1995). Osteoarthritis of the knee after injury to the anterior cruciate ligament or meniscus: the influence of time and age. Osteoarthritis Cartilage.

[5] Shrive, N. G., O’Connor, J. J. & Goodfellow, J. W. Load-bearing in the knee joint. *Clin Orthop Relat Res* 279-287 (1978). 657636

[6] Jaspers, P., De Lange, A. & Huiskes, R. The mechanical function of ~he meniscus, experiments on cadaveric pig knee-joints. (1980). 6894661

[7] Schumacher BL, Schmidt TA, Voegtline MS, Chen AC, Sah RL (2005). Proteoglycan 4 (PRG4) synthesis and immunolocalization in bovine meniscus. Journal of orthopaedic research.

[8] Verdonk PC (2005). Characterisation of human knee meniscus cell phenotype. Osteoarthritis Cartilage.

[9] Melrose J, Smith S, Cake M, Read R, Whitelock J (2005). Comparative spatial and temporal localisation of perlecan, aggrecan and type I, II and IV collagen in the ovine meniscus: an ageing study. Histochemistry and cell biology.

[10] Kambic HE, McDevitt CA (2005). Spatial organization of types I and II collagen in the canine meniscus. Journal of Orthopaedic Research.

[11] Eyre DR, Wu J-J (1983). Collagen of fibrocartilage: a distinctive molecular phenotype in bovine meniscus. FEBS letters.

[12] Arnoczky SP, Warren RF (1983). The microvasculature of the meniscus and its response to injury. An experimental study in the dog. Am J Sports Med.

[13] Arnoczky SP, Warren RF (1982). Microvasculature of the human meniscus. Am J Sports Med.

[14] McDermott ID, Amis AA (2006). The consequences of meniscectomy. J Bone Joint Surg Br.

[15] Niu, W. *et al*. Cell-Based Strategies for Meniscus Tissue Engineering. *Stem cells international* **2016** (2016). 10.1155/2016/4717184 PMC4871968 27274735

[16] Marsano A (2007). Differential cartilaginous tissue formation by human synovial membrane, fat pad, meniscus cells and articular chondrocytes. Osteoarthritis Cartilage.

[17] Marsano A (2006). Use of hydrodynamic forces to engineer cartilaginous tissues resembling the non-uniform structure and function of meniscus. Biomaterials.

[18] Pabbruwe MB (2010). Repair of meniscal cartilage white zone tears using a stem cell/collagen-scaffold implant. Biomaterials.

[19] Horie M (2012). Implantation of allogenic synovial stem cells promotes meniscal regeneration in a rabbit meniscal defect model. The Journal of bone and joint surgery. American volume.

[20] Ruiz-Ibán MÁ (2011). The effect of the addition of adipose-derived mesenchymal stem cells to a meniscal repair in the avascular zone: an experimental study in rabbits. Arthroscopy: The Journal of Arthroscopic & Related Surgery.

[21] Ding Z, Huang H (2015). Mesenchymal stem cells in rabbit meniscus and bone marrow exhibit a similar feature but a heterogeneous multi-differentiation potential: superiority of meniscus as a cell source for meniscus repair. BMC Musculoskelet Disord.

[22] Shen W (2014). Intra-articular injection of human meniscus stem/progenitor cells promotes meniscus regeneration and ameliorates osteoarthritis through stromal cell-derived factor-1/CXCR4-mediated homing. Stem Cells Transl Med.

[23] McCorry MC, Bonassar LJ (2017). Fiber development and matrix production in tissue-engineered menisci using bovine mesenchymal stem cells and fibrochondrocytes. Connective tissue research.

[24] Pelttari K (2006). Premature induction of hypertrophy during *in vitro* chondrogenesis of human mesenchymal stem cells correlates with calcification and vascular invasion after ectopic transplantation in SCID mice. Arthritis Rheum.

[25] Herwig J, Egner E, Buddecke E (1984). Chemical changes of human knee joint menisci in various stages of degeneration. Annals of the rheumatic diseases.

[26] Nakata, K. *et al*. Human meniscus cell: characterization of the primary culture and use for tissue engineering. *Clin Orthop Relat Res* S208-218 (2001). 11603705

[27] Gunja NJ, Athanasiou KA (2007). Passage and reversal effects on gene expression of bovine meniscal fibrochondrocytes. Arthritis research & therapy.

[28] Croutze R, Jomha N, Uludag H, Adesida A (2013). Matrix forming characteristics of inner and outer human meniscus cells on 3D collagen scaffolds under normal and low oxygen tensions. BMC musculoskeletal disorders.

[29] Son, M. S. & Levenston, M. E. Quantitative tracking of passage and 3D culture effects on chondrocyte and fibrochondrocyte gene expression. *J Tissue Eng Regen Med*, 10.1002/term.2022 (2015). 10.1002/term.2022 25824488

[30] Stevens MM, Marini RP, Martin I, Langer R, Shastri VP (2004). FGF‐2 enhances TGF‐β1‐induced periosteal chondrogenesis. Journal of orthopaedic research.

[31] Cui X, Breitenkamp K, Lotz M,  D’Lima D (2012). Synergistic action of fibroblast growth factor‐2 and transforming growth factor‐beta1 enhances bioprinted human neocartilage formation. Biotechnol. Bioeng..

[32] Jakob M (2001). Specific growth factors during the expansion and redifferentiation of adult human articular chondrocytes enhance chondrogenesis and cartilaginous tissue formation *in vitro*. J Cell Biochem.

[33] Adesida AB, Mulet-Sierra A, Laouar L, Jomha NM (2012). Oxygen tension is a determinant of the matrix-forming phenotype of cultured human meniscal fibrochondrocytes. PLoS One.

[34] Adesida AB, Grady LM, Khan WS, Hardingham TE (2006). The matrix-forming phenotype of cultured human meniscus cells is enhanced after culture with fibroblast growth factor 2 and is further stimulated by hypoxia. Arthritis Res Ther.

[35] Solchaga LA, Penick K, Goldberg VM, Caplan AI, Welter JF (2010). Fibroblast growth factor-2 enhances proliferation and delays loss of chondrogenic potential in human adult bone-marrow-derived mesenchymal stem cells. Tissue Eng Part A.

[36] Solchaga LA (2005). FGF-2 enhances the mitotic and chondrogenic potentials of human adult bone marrow-derived mesenchymal stem cells. Journal of cellular physiology.

[37] Martin I, Vunjak-Novakovic G, Yang J, Langer R, Freed L (1999). Mammalian chondrocytes expanded in the presence of fibroblast growth factor 2 maintain the ability to differentiate and regenerate three-dimensional cartilaginous tissue. Experimental cell research.

[38] Barbero A, Ploegert S, Heberer M, Martin I (2003). Plasticity of clonal populations of dedifferentiated adult human articular chondrocytes. Arthritis & Rheumatism.

[39] Guerne PA, Sublet A, Lotz M (1994). Growth factor responsiveness of human articular chondrocytes: distinct profiles in primary chondrocytes, subcultured chondrocytes, and fibroblasts. Journal of Cellular Physiology.

[40] Pennathur-Das R, Levitt L (1987). Augmentation of *in vitro* human marrow erythropoiesis under physiological oxygen tensions is mediated by monocytes and T lymphocytes. Blood.

[41] Lund‐Olesen K (1970). Oxygen tension in synovial fluids. Arthritis & Rheumatism.

[42] Bornes TD, Adesida AB, Jomha NM (2014). Mesenchymal stem cells in the treatment of traumatic articular cartilage defects: a comprehensive review. Arthritis research & therapy.

[43] Leijten J (2014). Metabolic programming of mesenchymal stromal cells by oxygen tension directs chondrogenic cell fate. Proceedings of the National Academy of Sciences.

[44] Falanga V, Zhou L, Yufit T (2002). Low oxygen tension stimulates collagen synthesis and COL1A1 transcription through the action of TGF-beta1. J Cell Physiol.

[45] Sophia Fox AJ, Bedi A, Rodeo SA (2009). The basic science of articular cartilage: structure, composition, and function. Sports Health.

[46] Mauck RL, Martinez‐Diaz GJ, Yuan X, Tuan RS (2007). Regional multilineage differentiation potential of meniscal fibrochondrocytes: implications for meniscus repair. The Anatomical Record.

[47] Crisan M (2008). A perivascular origin for mesenchymal stem cells in multiple human organs. Cell stem cell.

[48] Segawa Y (2009). Mesenchymal stem cells derived from synovium, meniscus, anterior cruciate ligament, and articular chondrocytes share similar gene expression profiles. Journal of Orthopaedic Research.

[49] Shen W (2013). Osteoarthritis prevention through meniscal regeneration induced by intra-articular injection of meniscus stem cells. Stem Cells Dev.

[50] Seol, D. *et al*. Characteristics of meniscus progenitor cells migrated from injured meniscus. *Journal of Orthopaedic Research* (2016). 10.1002/jor.23472 PMC6354255 27813166

[51] Hellio Le Graverand MP (2001). The cells of the rabbit meniscus: their arrangement, interrelationship, morphological variations and cytoarchitecture. J Anat.

[52] Fu, W. *et al*. Isolation, Characterization, and Multipotent Differentiation of Mesenchymal Stem Cells Derived from Meniscal Debris. *Stem cells international* **2016** (2016). 10.1155/2016/5093725 PMC5164906 28044083

[53] Adesida AB, Mulet-Sierra A, Jomha NM (2012). Hypoxia mediated isolation and expansion enhances the chondrogenic capacity of bone marrow mesenchymal stromal cells. Stem Cell Res Ther.

[54] Sun Y (2010). Analysis of meniscal degeneration and meniscal gene expression. BMC musculoskeletal disorders.

[55] Barbero A (2004). Age related changes in human articular chondrocyte yield, proliferation and post-expansion chondrogenic capacity. Osteoarthritis and cartilage.

[56] Takroni T, Laouar L, Adesida A, Elliott JA, Jomha NM (2016). Anatomical study: comparing the human, sheep and pig knee meniscus. Journal of Experimental Orthopaedics.

[57] Bornes TD, Jomha NM, Mulet-Sierra A, Adesida AB (2016). Optimal Seeding Densities for *In Vitro* Chondrogenesis of Two- and Three-Dimensional-Isolated and -Expanded Bone Marrow-Derived Mesenchymal Stromal Stem Cells Within a Porous Collagen Scaffold. Tissue Eng Part C Methods.

[58] Lee CH (2014). Protein-releasing polymeric scaffolds induce fibrochondrocytic differentiation of endogenous cells for knee meniscus regeneration in sheep. Science translational medicine.

[59] Martin I, Vunjak-Novakovic G, Yang J, Langer R, Freed LE (1999). Mammalian chondrocytes expanded in the presence of fibroblast growth factor 2 maintain the ability to differentiate and regenerate three-dimensional cartilaginous tissue. Exp Cell Res.

[60] Bornes TD, Jomha NM, Mulet-Sierra A, Adesida AB (2015). Hypoxic culture of bone marrow-derived mesenchymal stromal stem cells differentially enhances *in vitro* chondrogenesis within cell-seeded collagen and hyaluronic acid porous scaffolds. Stem cell research & therapy.

[61] Farndale RW, Buttle DJ, Barrett AJ (1986). Improved quantitation and discrimination of sulphated glycosaminoglycans by use of dimethylmethylene blue. Biochim Biophys Acta.

[62] Livak KJ, Schmittgen TD (2001). Analysis of relative gene expression data using real-time quantitative PCR and the 2−ΔΔCT method. methods.

